# Identification of early molecular markers for breast cancer

**DOI:** 10.1186/1476-4598-10-15

**Published:** 2011-02-11

**Authors:** Céline Kretschmer, Anja Sterner-Kock, Friederike Siedentopf, Winfried Schoenegg, Peter M Schlag, Wolfgang Kemmner

**Affiliations:** 1 Research Group Surgical Oncology, ECRC, Robert-Rössle-Str. 10, 13125 Berlin, Germany; 2 Center for Experimental Medicine, University of Cologne, Medical School, Robert Kochstr. 10, 50931 Cologne, Germany; 3 DRK Kliniken Berlin Westend, Brustzentrum, Spandauer Damm 130, 14050 Berlin, Germany; 4 Charité Comprehensive Cancer Center, Charite Campus Mitte, Invalidenstrasse 80, 10117 Berlin, Germany

## Abstract

**Background:**

The ductal carcinoma *in situ *(DCIS) of the mammary gland represents an early, pre-invasive stage in the development of invasive breast carcinoma. Since DCIS is a curable disease, it would be highly desirable to identify molecular markers that allow early detection. Mice transgenic for the WAP-SV40 early genome region were used as a model for DCIS development. Gene expression profiling was carried out on DCIS-bearing mice and control animals. Additionally, a set of human DCIS and invasive mammary tumors were analyzed in a similar fashion. Enhanced expression of these marker genes in human and murine samples was validated by quantitative RT-PCR. Besides, marker gene expression was also validated by immunohistochemistry of human samples. Furthermore *in silico *analyses using an online microarray database were performed.

**Results:**

In DCIS-mice seven genes were identified that were significantly up-regulated in DCIS: DEPDC1, NUSAP1, EXO1, RRM2, FOXM1, MUC1 and SPP1. A similar up-regulation of homologues of the murine genes was observed in human DCIS samples. Enhanced expression of these genes in DCIS and IDC (invasive ductal carcinoma) was validated by quantitative RT-PCR and immunohistochemistry.

**Conclusions:**

By comparing murine markers for the ductal carcinoma *in situ *(DCIS) of the mammary gland with genes up-regulated in human DCIS-samples we were able to identify a set of genes which might allow early detection of DCIS and invasive carcinomas in the future. The similarities between gene expression in DCIS and invasive carcinomas in our data suggest that the early detection and treatment of DCIS is of utmost relevance for the survival of patients who are at high risk of developing breast carcinomas.

## Background

Early diagnosis and administration of effective treatment is the best strategy to combat cancer [1]. Starting in the early 1980 s, the increasing use of mammography screens has resulted in an increase in diagnosis of the ductal carcinoma *in situ *(DCIS), especially among women more than 50 years of age [2]. DCIS represents 20-45% of all new cases of mammographically detected breast cancer, and about 10% of all breast carcinomas [3]. Up to 50% of DCIS lesions progress to invasive breast cancer, but there is tremendous variability in the time of progression to invasive disease [4]. Today most DCIS cases are identified as suspicious microcalcifications through mammography. However, the accuracy of mammography in diagnosing DCIS is suboptimal [4]. The main drawback with respect to DCIS is that mammography often underestimates both the pathologic extent of DCIS and the number of tumour foci in patients with multifocal disease [2]. Early detection of DCIS is very important because it is a highly curable disease, with a 10-year cancer-specific survival rate of over 97% [3].

Therefore, biomarkers for DCIS are needed. In many types of carcinomas, biomarkers have enhanced our ability for diagnosis, prognosis, and for therapy prediction. In general, an appropriate biomarker should be useful in defining risks and identifying the early stages of carcinogenesis. Furthermore, biomarkers can be analyzed in a noninvasive and economic way and therefore it is worth investing in the search for more biomarkers [5].

The use of microarray technologies for gene expression profiling provides insight into the molecular basis of DCIS. Only a few gene expression profiling studies of DCIS have been published to date and most focus on the identification of progression-associated genes by comparison of *in situ *and invasive disease [6-8]. Gene expression profiling of DCIS is hindered by the limited numbers of samples available. To overcome the latter problem, our study used a transgenic mouse model for DCIS [9]. Mice were transgenic for the WAP-SV40 early genome region, so that expression of the SV40 oncogene is activated by lactation. The use of these transgenic animals offers the possibility of determining tumour-initiating factors and investigating gene expression at different stages of tumour development.

In the present work, we identified molecular markers for the ductal carcinoma *in situ*. Marker genes identified in the WAP-TNP8 mouse model were further investigated in a small human DCIS cohort. Identification of markers for DCIS and early invasive tumours is important for early detection and the development of improved therapeutic strategies.

## Materials and methods

### Mice

WAP-TNP8 animals, which selectively synthesize the T/t-antigen under the control of the WAP promoter in mammary gland epithelial cells, were used for this study [9]. In these mice the SV40 large tumour antigen is specifically induced by lactation. As a consequence of continuous expression of the oncogene, the animals develop multifocal DCIS and consequently invasive carcinoma. In general, the SV40-Tag system has very well documented intraluminal lesions which have been thoroughly analyzed with histology, immunohistochemistry, whole mounts and electron microscopy. These early lesions are typically solid masses of poorly differentiated cells with relatively compact hyperchromatic nuclei and scanty cytoplasm. They resemble some forms of human intraductal carcinomas [10]. WAP-TNP8 mice show rapidly growing, palpable tumours which are evident on average 4 months after induction. DCIS lesions of the transgenic mice exhibit distinct architectural and cytological features which closely resemble those commonly present in humans. The tumours mostly display a poorly differentiated solid or even anaplastic morphology, well differentiated tumours are rarely found. More precisely, WAP-T-NP8 mice show cribriform morphology of in situ carcinoma [9].

Wildtype mice and transgenic mice before lactation were used as negative controls, so that changes simply related to the transgenic profile could be ruled out. Mice were analysed one month after lactation (abbreviated as 1 m), two months after lactation (2 m), three months after lactation (3 m), four months after lactation (4 m) and five months after lactation (5 m). In this way we were able to study the development of DCIS at different time points. Similarly, invasive ductal carcinomas (IDC) were investigated and served as a positive control. Invasive tumors were obtained from mice taken at 4 or 5 months after lactation. Each group consisted of at least seven mice. For subsequent analysis, mice were sacrificed and mammary glands were dissected. From each mouse four milk ducts were prepared. One part of each mammary gland was cryopreserved in liquid nitrogen and stored at -80°C for RNA preparation and another part was fixed overnight in 5% formaldehyde and embedded in paraffin.

### Human tissue

Nineteen freshly frozen human breast tumour samples were obtained from the Robert-Rössle-Biobank at the ECRC (Experimental and Clinical Research Center). Tissue samples were cryopreserved immediately after surgery in liquid nitrogen and stored at -80°C. All participants have given written, informed consent. The study was approved by the local ethics committee (Charité Universitätsmedizin Berlin). The patient cohort consisted of nine DCIS, five invasive ductal carcinoma (IDC) and five healthy control samples obtained from patients with breast reduction surgery. A second panel consisting of human formalin-fixed paraffin-embedded (FFPE) tissue samples was used for immunohistochemical stainings. The panel consisted of 5 healthy, 10 DCIS and 5 IDC. DCIS samples were distinguished according to their grade (5 low grade DCIS/5 high grade DCIS). All samples were reviewed for histological classification according to nuclear grade and classified as low, intermediate, and high nuclear grade; additionally, the TNM-Stage and hormone receptor status were determined.

### RNA isolation, amplification and microarray analysis

RNA extraction from murine samples was performed using Qiagen RNeasy mini kit (Qiagen, Hilden, Germany) with on column DNAse I digestion in accordance with the manufacturer's guide. Human RNA was isolated with RNeasy Lipid Tissue Mini Kit (Qiagen). RNA quality was checked on Agilent 2100 Bioanalyzer (Agilent Technologies, Böblingen, Germany). For further analysis only samples with a RIN (RNA integrity number) of more than seven were taken.

Two-round linear amplification, using 50 ng total RNA, was carried out for the murine samples according to the GeneChip^® ^Two-Cycle Target Labelling protocol (Affymetrix, Santa Clara, CA, USA). In human samples cRNA was amplified from 1 μg of total RNA using the GeneChip^® ^One-Cycle Target Labelling Kit (Affymetrix). Quantities of *in vitro *transcription and fragmentation products were assessed using the Agilent 2100 Bioanalyzer. Labelled and fragmented cRNA was hybridized for 16 h at 45°C on Affymetrix oligonucleotide Murine Genome 430 2.0 or Human Genome U133 plus 2.0 Arrays. Hybridized arrays were scanned using the GeneChip Scanner 3000.

### Statistical analysis

An initial analysis was performed using the Affymetrix Microarray Suite 5.0 (MAS5) software. The percentage of present calls, background noise, the scaling factor, and the ratio of 3' to 5' hybridization for GAPDH and β-actin were used to assess quality of hybridization. Raw image data were converted to CEL files using the Affymetrix GeneChip Operating Software (GCOS). For adjacent analyses of microarray data, the GeneSpring GX 10.0 Software (Agilent Technologies) was used. GCRMA (GC robust multiarray average) was used to perform background correction and normalization. The mouse data is deposited as GEO series GSE21444, http://www.ncbi.nlm.nih.gov/geo/query/acc.cgi?token=btetzoskmeoguzg&acc=GSE21444, and the human as GSE21422, http://www.ncbi.nlm.nih.gov/geo/query/acc.cgi?token=lhsfdsoicaekcho&acc=GSE21422.

In order to identify differentially expressed genes between controls and samples taken at early time points (month 2-3 after lactation), as well as between controls and tumours, probe sets were filtered using the Welch-Test (unpaired T-test; unequal variance) with Benjamini and Hochberg False Discovery Rate. The fold-change threshold was 5.0 and the corrected p-value was set to ≤ 0.01. Volcano Plots visualize all probe sets according to corrected p-value and fold change. Using a Venn diagram, probe sets present in both lists were selected. The annotations of each probe set were obtained from the Affymetrix's NetAffx™ database. Two-dimensional unsupervised and supervised hierarchical clustering using Euclidean distance as distance function and complete linkage were performed. This method groups samples on the basis of similarity in their expression pattern.

### Quantitative RT-PCR

Quantitative RT-PCR was performed using TaqMan^® ^Gene Expression Assays and the ABI Prism™ 7900 HT Sequence Detection System (Applied Biosystems, Foster City, CA, USA). Gene Expression Assay IDs are listed in additional file 1 (Table S1 +S2). For the murine samples, the RNA UltraSense™ One-Step Quantitative RT-PCR System (Invitrogen, Carlsbad, CA, USA) was used. The procedure was performed in accordance with the manufacturer's guide. For human RNA, cDNA synthesis was done using Oligo(dT) primers and SuperScript II. For the relative quantification of gene expression, triplicate reactions were conducted. The expression of β-actin served as an internal control because β-actin expression levels were consistent throughout all samples through the cDNA microarray data. Relative expression was calculated according to the ΔΔCt method [11] using an internal reference sample as calibrator.

### Immunohistochemistry and H&E staining

Thin paraffin sections of the murine mammary glands (2-4 μm) were stained with haematoxylin and eosin according to standard procedures and histomorphologically evaluated by light microscopy. After deparaffinisation and rehydration, human tissue samples were boiled in citrate buffer (pH 6.0) for 5 min. Endogenous peroxidase was blocked using the DAKO Biotin Blocking System (DAKO, Glostrup, Denmark). Primary antibodies (additional file 1, Table S3) were mostly applied (1:100) for 1-2 h at room temperature. For each antibody, internal and external controls were included in the experiments. In negative controls the primary antibody was omitted. Sites of antigen-antibody binding were detected using biotinylated anti-mouse/rabbit/goat antibodies (Vector Laboratories, Burlingame, CA, USA). The chromogen used was Neufuchsin (Merck, Darmstadt, Germany). Slides were counterstained with haematoxylin and after dehydration were mounted in Entellan.

For each protein multiple immunohistochemical stainings were performed (5 healthy,5 low grade DCIS, 5 high grade DCIS and 5 IDC). A semi-quantitative scoring system was used for the evaluation of the immunohistochemical staining (Table 1). Figures show representative pictures.

**Table 1:** Staining pattern of the immunohistochemical analysis of different human mammary tissue samples using a semi-quantitative scoring system

	healthy control	low grade DCIS	high grade DCIS	IDC
MUC1	-	+++	+++	+++
SPP1	-/+	+++	+++	+++
RRM2	-	+++	+++	+++
FOXM1	+	+++	+++	+++
DEPDC1	+	+++	+++	++++
NUSAP1	-	+++	+++	+++

- = no imunoreactive cells, + = 1-5 immunoreactive cells, ++ = 5-10 immunoreactive cells, +++ = 10-100 immunoreactive cells, ++++ = >100 immunoreactive cells. Average number of cells per high power field is given, 5 high power fields were evaluated.

## Results

### Identification of murine DCIS markers

Gene expression patterns of control samples, of samples taken at different time points after lactation, and of invasive breast tumours (IDC) from 40 mice (five samples per group) were analysed. Animals examined one month after activation of the oncogene were excluded from further analysis because of artifacts due to lactation. Histological investigations of all groups were performed. The majority of DCIS arises by month three or later.

First a t-test was conducted comparing the control groups (wild type mice + mice before lactation) with mice taken two and three months after lactation. This comparison revealed 230 probe sets which are differentially expressed between control samples and mice in which the development of DCIS had already been induced. A second t-test was conducted in order to compare controls and invasive mammary tumours. This procedure resulted in a list of 2398 probe sets which were differentially expressed between controls and invasive mammary tumours. To obtain tumour-specific genes that are already up-regulated in DCIS, only genes present in both lists were used for further analysis. A total of 173 probe sets met these criteria and were considered as potential candidate genes for early DCIS detection. These 173 probe sets cover 140 genes (additional file 1, Table S4).

**Figure 1:**
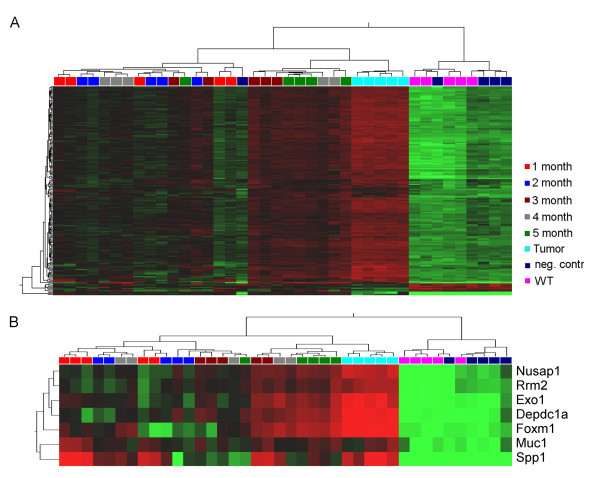
**Microarray analysis of murine samples**. **A**: Supervised hierarchical clustering using 173 probe sets (= 140 genes) overexpressed in mice taken 2-3 months after lactation and in IDC of WAP-TNP8 mice. Each row represents a probe set and each column a sample. The length and the subdivision of branches display the relation of the samples based on their expression. Each group contains samples obtained from five mice. The time point of determination of gene expression was calculated as months after lactation (1 month, 2 months,...). As a positive control IDCs (Tumor) were used. Additionally, wild type (WT = Balb/C) mice and mice without lactation (neg. contr) were used as negative controls. Red indicates upregulation, green downregulation, and black no change. **B**: Supervised hierarchical clustering of the murine samples using the seven marker genes clearly distinguishes between control samples and malignant samples.

Supervised hierarchical clustering using the 140 candidate genes revealed tight clustering of murine samples of the same month after lactation (Figure 1A). The vast majority of the 140 candidate genes were up-regulated in DCIS and tumour samples. As the pattern and length of the branches reflects the relatedness of the samples, these 140 genes clearly distinguish between control samples and malignant samples. Besides, it is obvious that the samples of the late time points after lactation (3 - 5 months) exhibited an expression of the 140 genes similar to that of invasive tumour samples.

In order to identify a minimal set of genes as final candidates, the distribution of the expression values of the 140 significantly changed candidate genes was investigated. Only genes showing a enhanced expression in the malignant samples were considered. Genes which showed constant up-regulation during DCIS-development and low variance within the groups were chosen as final marker genes. These are: MUC1, SPP1, RRM2, FOXM1, EXO1, NUSAP1 and DEPDC1. Using these seven genes for supervised hierarchical clustering allowed us to separate healthy control samples from all other samples. Again, the tumour samples clustered in the same branch as most of the samples of the late time points (3, 4 and 5 months) (Figure 1B).

**Figure 2:**
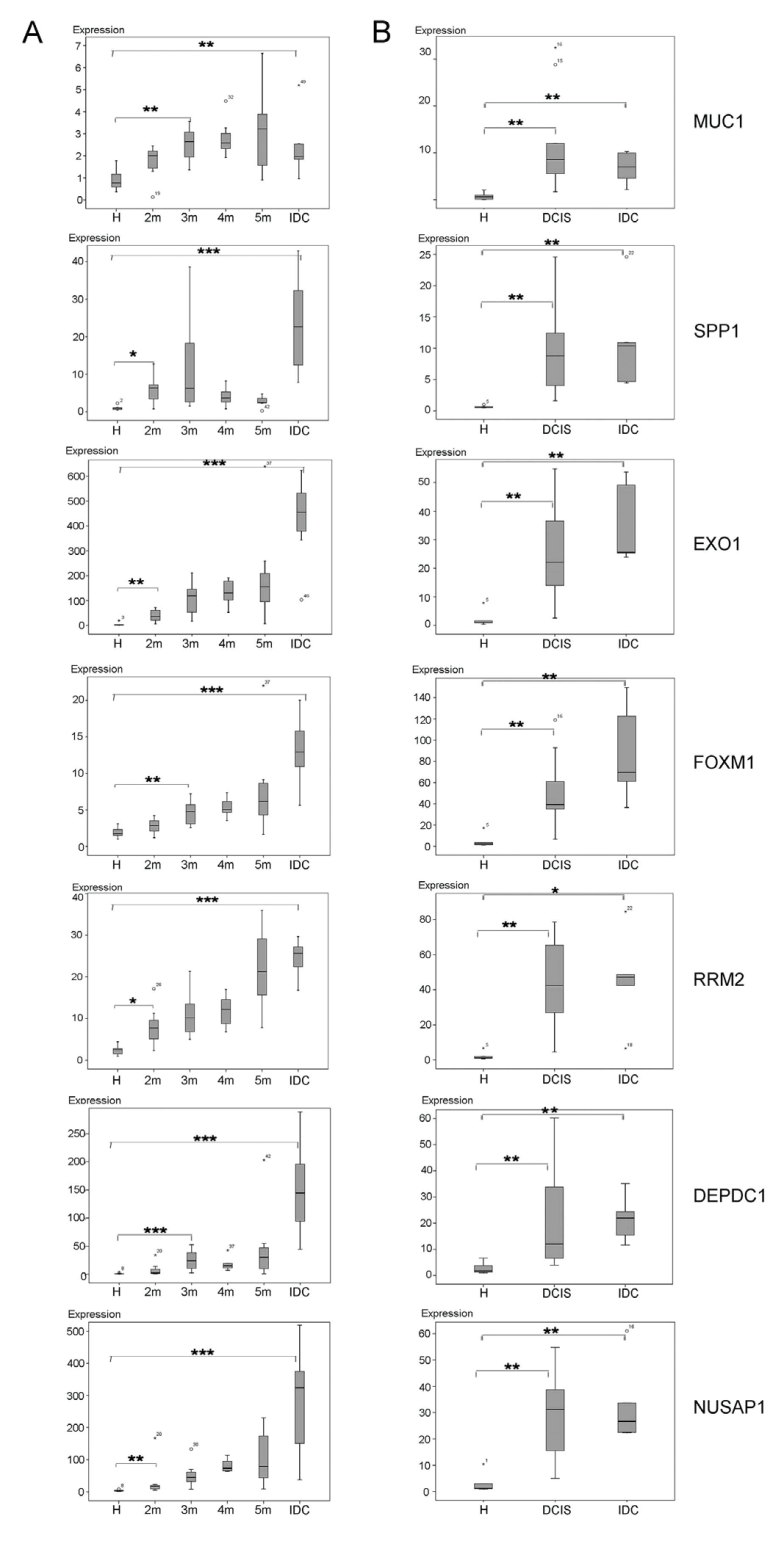
**Validation of the marker gene expression by RT-PCR**. Relative expression is shown in Box - Whisker - Plots. Gray columns show a 50% range of the data surrounding the median; black lines within each column mark the median; circles mark outliers. Significance was calculated with the Mann-Whitney-U test (P < = 0.05*, P < = 0.01**, P < = 0.001 three stars). **A**: Panel of the murine samples. Controls are transgenic mice before lactation (H). Months are calculated from the start of lactation (2 m = 2 months; 3 m = 3 months; 4 m = 4 months; 5 m = 5 months; IDC = invasive ductal carcinoma). Each group contains 7 samples. **B**: Panel of human samples. Controls are healthy tissues from reduction plastics (H).

To confirm the microarray results, the expression of the seven marker genes was validated by quantitative RT-PCR (Figure 2A). Each group consisted of seven murine samples. Results confirmed very well the findings of the microarray analysis. A comparison of microarray and qRT-PCR box plots showed nearly identical pictures, hence only the RT-PCR results are shown here. With the exception of two cases, the expression of the marker genes was already significantly up-regulated two months after lactation, although in histological investigations almost no DCIS was found. In the case of FOXM1 and DEPDC1 up-regulation in month two was not significant, but that had changed by month three. In most of the genes there was a continuous increase of expression which reached the highest point in the IDC.

### Analysis of human DCIS samples

**Figure 3:**
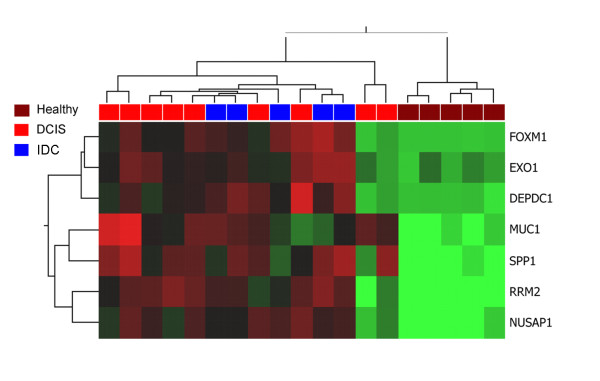
**Microarray analysis of human samples**. Supervised hierarchical clustering using of the human samples using the seven marker gene set clearly distinguishes between control samples and malignant samples. Each row represents a probe set and each column a sample. Red indicates upregulation, green downregulation, and black no change.

As a next step we investigated the gene expression of human DCIS samples. To this end we used a set of 19 samples consisting of five healthy controls, five invasive tumours and nine DCIS samples. Expression profiles were recorded by Affymetrix U133 plus 2.0 GeneChips. An unsupervised hierarchical clustering of the human samples shows the healthy samples separated from the DCIS and IDC samples. The DCIS samples showed a comparative expression profile similar to that of the invasive breast carcinomas (Data not shown). The human data were analyzed in the same fashion as the murine samples. However, we focused on the markers found already in the murine analysis. Statistical analysis revealed a strong up-regulation of the seven previously identified marker genes in human DCIS as well. This led us to conclude that the marker genes can be used as early detection markers also for human DCIS. Hierarchical clustering using these seven genes showed that DCIS and invasive carcinomas were clearly separated from healthy samples (Figure 3). Within the malignant branch DCIS and invasive carcinomas could not be distinguished.

We also analysed genes which were significantly up-regulated only in DCIS but not in IDC. In the murine samples no such genes could be identified. In the human samples 5 genes were found which showed significant up-regulation in DCIS but not in IDC in comparison to healthy samples. The most interesting gene was WNT5A. Recent work in a wide range of human tumours has pointed to a critical role for the Wnt signaling molecule Wnt-5a in malignant progression, but there is conflicting evidence whether Wnt-5a has a tumour-promoting or -suppressing role [12]. Expression of WNT5A was not further investigated in the present contribution.

Microarray results for the seven candidate genes described above were validated by quantitative PCR. Expression differences were highly significant between healthy controls and DCIS samples (Figure 2B). In table 2 the most important reported functions of each of the seven marker genes are depicted.

**Table 2:** Overview of the main features of the candidate genes. Human Entrez Gene ID is shown in the last column.

Symbol	Name	Go terms: biological process; molecular function	GO terms: cellular component	Entrez GeneID
MUC1	mucin 1, cell surface associated	hormone activity	extracellular region, nucleus, cytoplasm, integral to membrane	4582
SPP1	secreted phosphoprotein 1	ossification, cell adhesion; cytokine activity, protein binding	extracellular region	6696
RRM2	ribonucleotide reductase M2 polypeptide	DNA replication, deoxyribonucleoside diphosphate metabolic process, oxidation reduction; ribonucleoside-diphosphate reductase activity, iron ion binding, protein binding, oxidoreductase activity	cytoplasm, cytosol	6241
FOXM1	forkhead box M1	regulation of transcription, DNA-dependent, vasculogenesis, positive regulation of cell proliferation; DNA binding, transcription factor activity, protein binding	nucleus	2305
EXO1	exonuclease 1	DNA repair, mismatch repair, DNA recombination, immune response, meiosis; DNA binding, catalytic activity, exonuclease activity, endonuclease activity, ribonuclease H activity, protein binding, hydrolase activity	nucleus	9156
DEPDC1	DEP domain containing 1	signal transduction, intracellular signaling cascade; GTPase activator activity	intracellular, nucleus	55635
NUSAP1	nucleolar and spindle associated protein 1	mitotic sister chromatid segregation, cell cycle, mitosis, establishment of mitotic spindle localization, cell division; DNA binding, microtubule binding	nucleus, cytoplasm, microtubule	51203

**Figure 4:**
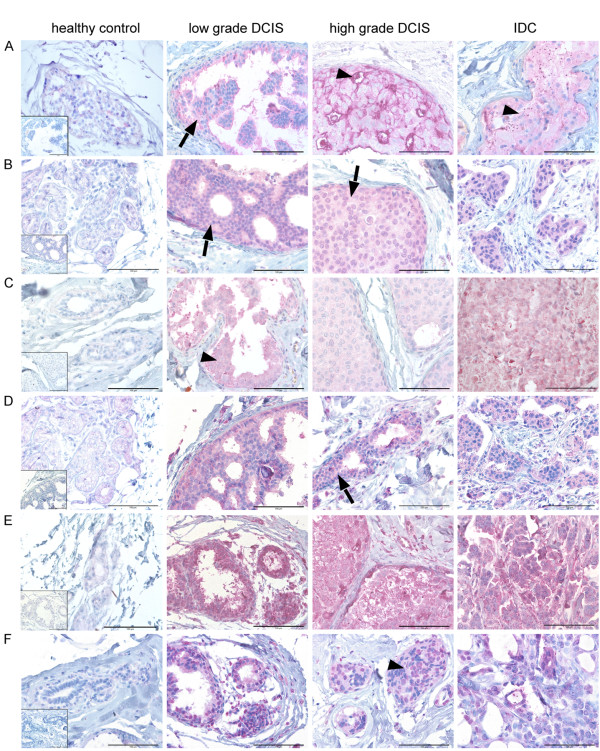
**Histological analysis of markers genes**. Protein expression was determined by immunohistochemistry using sections from Formalin-fixed paraffin-embedded tissue **(A**: MUC1, **B**: SPP1, **C**: RRM2, **D**: FOXM1, **E**: DEPDC1, **F**: NUSAP1). For each protein, expression is shown in human breast tissue with a rising degree of malignancy (healthy, DCIS, invasive breast tumour). Specific signals are represented by pink staining (arrowhead) (counterstained with haematoxylin, original magnification 400×, bars:100 μm). The inserts depict the negative controls as a reference.

In order to further investigate the expression of these candidate genes at the cellular level *in vivo*, we performed immunohistochemical analyses in a panel of healthy human mammary gland tissue samples, DCIS and invasive breast tumours. To do so we used another set of formalin-fixed paraffin-embedded human tissue samples. For each protein multiple immunohistochemical stainings were performed (five samples per group). Representative examples are shown in figure 4. For EXO1 no specific antibody was found. Immunoreaction of the marker genes in healthy tissues was negative or very weak. However, immunoreaction in DCIS and IDC samples in the majority of cases was very intense. The expression of the protein was indicated by pink staining (exemplarily see arrowhead). Positive staining was predominantly visible within the lumina of the ducts, predominantly epithelial cells showed a positive signal (See arrows for examples). A positive staining was already visible in the low grade DCIS samples. The staining pattern was cytoplasmatic for SPP1, RRM2, FOXM1, DEPDC1 and NUSAP1. Membranous as well as cytoplasmatic staining was visible for MUC1.

## Discussion

The identification of gene expression signatures or molecular markers in DCIS is hindered by difficulties in obtaining sufficient numbers of frozen DCIS-samples from the hospital. Thus, we first approached the problem using a mouse model. We choose the WAP-TNP8 mouse model of Schulze-Garg et al. [9] because it is a well described model for DCIS and exhibits long latency in developing invasive tumours. This animal model has been used for detection of different tumour growth kinetics by flat-panel volume computed tomography [13], for the analysis of cell type-specific expression of Casein kinase 1 epsilon (CK1e) [14] and for a molecular imaging study of extradomain-b fibronectin (EDB-FN) targeting neoangiogenesis by near-infrared fluorescence [15]. In our study, we used this model for determining tumour-initiating factors and investigating gene expression profiles at different stages of tumour development. Gene profiling was confirmed within two panels of human DCIS samples. A panel of fresh frozen human samples was used for another gene expression profiling analysis in order to verify whether the expression of the marker genes identified in the murine samples agrees with that found in the human samples. A second panel of human FFPE samples, including high but also low grade DCIS, was used for a validation of the expression of the candidate genes on the protein level.

In this study, we identified seven marker genes which are overexpressed in DCIS and invasive carcinomas and allowed us to distinguish between healthy and DCIS samples. Our marker genes include MUC1, SPP1, RRM2, FOXM1, EXO1, NUSAP1 and DEPDC1. Some of these markers are already known to be related to DCIS; others are completely novel for DCIS and even for breast cancer. In the future, such molecular markers may allow an early detection of DCIS.

Epithelial mucin 1 (MUC1) is an accepted serum tumour marker and cellular tumour antigen [16]. According to immunohistological studies MUC1 protein expression is particular high in tumours, where it undergoes changes in glycosylation and distribution [17]. However a low level of expression of MUC1 is also found in healthy, undifferentiated (non-lactating) breast tissue [18]. The correlation between MUC1 expression and the clinical outcome of the patients is still under debate. While some *in-vitro *studies showed that MUC1 overexpression promotes cellular invasion [19,20] investigations of MUC1 expression of breast carcinomas have shown a better outcome for patients overexpressing MUC1 [21]. MUC1 was found to be commonly up-regulated in both DCIS and IDC [7]. Our results also confirmed earlier findings showing that MUC1 is also up-regulated on the protein level in DCIS [22].

Similarly, overexpression of Osteopontin (SPP1) has been found in a variety of cancers, including breast, lung, colorectal, stomach, ovarian cancers and melanoma [5,23]. SPP1 is a phosphorylated glycoprotein secreted by several cell types, including those involved in bone turnover and cells of the immune system [5,24]. SPP1 has been associated with breast cancer progression, invasion and metastasis [24-29] and is present in elevated levels in the blood and plasma of some patients with metastatic cancers [5]. We have found SPP1 to be significantly up-regulated in DCIS. Previously, Reinholz et al. investigated the expression of SPP1 in normal, non-invasive, invasive and metastatic human breast cancer specimens by RT-PCR [30]. They showed that the mRNA level of SPP1 increased in non-invasive, invasive and metastatic breast tumour tissue compared to normal breast tissue. We found an increase in staining intensity for SPP1 in DCIS samples compared to healthy controls, which confirms a study by Oyama et al., who detected positive staining of SPP1 using immunohistochemistry on paraffin-embedded tissues in most cases of low-grade cribiform and high-grade comedo-type ductal carcinoma *in situ *[31].

RRM2, a ribonucleotid reductase (RR), was shown to be overexpressed in human breast carcinoma tissue (DCIS) [32]. RR is responsible for the de novo conversion of ribonucleoside diphosphates to deoxyribonucleoside diphosphates that are essential for DNA synthesis and repair [33,34]. RR consists of two subunits, M1 (RRM1) and M2 (RRM2). It is known that alterations in RR levels can have significant effects on the biological properties of cells, including tumour promotion and tumour progression. In our findings, RRM2 was significantly up-regulated on the RNA as well as on the protein level.

Likewise, the transcription factor forkhead box M1 (FOXM1) was found to be differentially expressed in most solid tumours [35]. FOXM1 stimulates proliferation and cell cycle progression by promoting entry into both S-phase and mitosis. In addition, it plays a role in the proper execution of mitosis. FOXM1 is implicated in the tumourigenesis of more than 20 types of human tumours and contributes to both tumour initiation and progression [36]. FOXM1 is broadly expressed in breast epithelial cell lines and seems to be significantly increased in transformed breast epithelial cell lines. Consistently, FOXM1 expression is specifically elevated in breast carcinomas [37]. Using immunohistochemistry, Bektas et al. analysed FOXM1 expression in human invasive breast carcinomas and normal breast tissues on a tissue microarray [38]. In contrast to what could be expected from GO-analysis (Table 2) they found a strong cytoplasmatic expression of the transcription factor FOXM1, resulting most likely from its strong overexpression. Additionally, using RT-PCR, FOXM1 was found to be overexpressed in breast cancer in comparison to normal breast tissue both on the RNA and protein level. Furthermore, FOXM1 was found to be overexpressed during progression from DCIS to invasive breast cancer [7]. Our findings confirm these results. FOXM1 was significantly overexpressed already on the DCIS level and was even higher expressed in IDC.

In contrast, overexpression of EXO1, NUSAP1 and DEPDC1 in IDC and DCIS had not yet been described. We found these genes significantly up-regulated in DCIS as well as in IDC. EXO1 (exonuclease 1) has been implicated in a multitude of eukaryotic DNA metabolic pathways that include DNA repair, recombination, replication, and telomere integrity. This makes EXO1 a logical target for mutation during oncogenesis [39]. However, Rassmussen et al. have shown high expression levels of human EXO1 transcripts in liver cancer cell lines and in colon and pancreas adenocarcinomas, but not in the corresponding non-neoplastic tissue [40]. This is a first hint that EXO1 is up-regulated in tumours. Nucleolar spindle-associated protein (NUSAP1) was identified in 2003 as a novel 55-kD vertebrate protein with selective expression in proliferating cells [41]. mRNA and protein levels of NUSP1 peak at the transition of G2 to mitosis and abruptly decline after cell division. Interestingly, NUSAP1 was found to be up-regulated in melanoma cells by gene expression profiling of a series of melanoma cell lines [42]. Proteins such as NUSAP that show little or no expression in G1 and G0 may be reliable histochemical markers for proliferation and might therefore be useful for cancer prognosis [41]. NUSAP1 expression was significantly increased in DCIS and IDC in our study and is therefore a promising new tumour marker. DEPDC1 (DEP domain containing 1) is also a newly detected gene. Kanehira et al. identified DEPDC1 as a novel gene that is highly overexpressed in bladder cancer samples, but not expressed in any human organs (heart, liver, kidney, lung) except the testis [43]. Our findings show that DEPDC1 is significantly up-regulated in DCIS and IDC. Preliminary results from a study of the functional relevance of DEPDC1 show that it seems to be an important gene for proliferation as well as for migration and invasion (C.S. manuscript in progress).

We found that the seven putative marker genes are strongly up-regulated in mice and in human DCIS samples. This reveals that the mouse model we used reflects human breast cancer development. Previously, Klein et al. [44] compared the expression profile of 24 human breast tumours and six WAP-SVT/t mice breast tumours. They found 597 genes which are overexpressed in breast cancer in mice [44]. Their list also contains DEPDC1, NUSAP1, MUC1, EXO1, and RRM2. Some of our marker genes have been described previously in human breast cancer. In a 22-gene signature investigated by Martin et al. [45], FOXM1 and RRM2 were included. This signature accurately predicts breast cancer outcome [45]. Additionally, Ma et al. developed a gene expression index for tumour grade in breast cancer patients which included RRM2 [6]. This is further evidence that the candidate genes we identified are important in tumour development.

Candidate genes were further validated using Oncomine http://www.oncomine.org, a database for online cancer gene expression analysis. In the data set of Richardson et al. which compared normal breast tissue with IDC, six of our seven marker genes are significantly up-regulated in IDC [46]. Additionally, also using Oncomine to search for the tumour grade and the prognostic impact, we found that all the marker genes except MUC1 were significant for prognosis in the calculation of this database. Using a p-value of 0.001 these genes are up-regulated in multiple expression analyses in patients with a poor prognosis. This is an indication that our panel of marker genes could also be useful as a prognostic tool. Looking at the tumour grade, all the genes except MUC1 and SPP1 were significantly up-regulated in samples with a high tumour grade in Oncomine. Thus, the marker genes might indicate a high grade of malignancy. One explanation for this could be that in the analysis of the human samples, we used predominantly samples with a high tumour grade. On the other hand, in the case of the murine samples, the specimens we investigated were from a very early time point, where no DCIS (or few) were pathologically found.

In accordance with recent gene expression studies, our data support the hypothesis that critical molecular events which have a profound influence on development, progression and outcome of human breast cancer occur at an early stage. Despite significant morphologic differences between the different stages, expression profiles of early lesions are highly similar to the more advanced, invasive lesions [47]. This has been demonstrated also on the protein level [48]. Sorlie et al. claimed that extensive studies of DCIS and other pre-invasive stages of tumours will enhance this hypothesis and substantiate the value of gene expression-based classification in the prognosis of breast cancer at an early stage [49]. Furthermore Ma et al. [50] showed that the tumour microenvironment of invasive breast tumours also participates in tumourigenesis even before tumour cells invade into stroma. This is a further hint that changes during breast cancer development occur at a very early time point and that also the tumour microenvironment plays an important role in the transition from preinvasive to invasive growth. We took a step in this direction by showing on the RNA level as well as on the protein level that the marker genes we found are already significantly up-regulated on the level of DCIS and likewise later on the IDC level.

## Conclusions

Summing up, we found seven putative tumour markers which are strongly expressed at a very early stage of premalignancy and preneoplasia of breast carcinomas. In the future, the identified marker genes might allow an early diagnosis of DCIS and thereby improve prognosis of breast cancer. One next step will be to couple specific probes for these marker genes to near-infrared-dyes and examine whether early lesions can be detected also in an in-vivo animal model.

## List of abbreviations

DCIS: ductal carcinoma *in situ; *DEPDC1: DEP domain containing 1; EXO1: exonuclease 1; FFPE: formalin-fixed paraffin-embedded; FOXM1: forkhead box M1; GCOS: Affymetrix GeneChip Operating Software; GCRMA: GC robust multiarray average; IDC: invasive ductal carcinoma; MAS5: Microarray Suite 5.0; MUC1: mucin 1; NUSAP1: Nucleolar spindle-associated protein; Oncomine: online Microarray database http://www.oncomine.org; RIN: RNA integrity number; RRM2: ribonucleotid reductase M2; RT-PCR: Real Time polymerase chain reaction; SPP1: Osteopontin; SV40: Simian Virus 40; TNM: Tumour, Nodes, Metastasis; WAP: Whey acidic protein; WNT5A: wingless-type MMTV integration site family, member 5A;

## Competing interests

The authors declare that they have no competing interests.

## Authors' contributions

CS carried out the laboratory work, carried out the statistical analyses, prepared the majority of the figures and wrote the manuscript. WK conceived the study and contributed to writing of the manuscript. ASK analyzed tumour samples and examined the IHC stainings. FS carried out sample collection. WS was responsible for collection of the samples, provided clinical information and critical revision of the manuscript. PMS participated in the discussion and critical revision of the manuscript. All authors read and approved the manuscript.

## Supplementary Material

Additional file 1 **Table S1. Assays on demand (Applied Biosystems) used for the human RT-PCR**. Table S1 gives an overview about the Assays on demand used for the RT-PCR on the human samples. **Table S2. Assays on demand (Applied Biosystems) used for the murine RT-PCR**. Table S2 gives an overview about the Assays on demand used for the RT-PCR on the murine samples. **Table S3. Primary antibodies used for immunhistochemical staining**. Table S3 gives an overview about the Antibodys used for the immunohistochemistry on the human tissue samples. The Table includes information about the dilution, the Company and the catalog number of the antibody. **Table S4. 173 probe sets significantly changed between controls and DCIS/IDC in WAP-TNP-8 mice**. Table S4 shows all the genes found to be differentially expressed between control mice and DCIS/IDC in the WAP-TNP8 mice. Click here for file
