# Supplementary material for: Identification of early molecular markers for breast cancer
Source: Mol Cancer. 2011 Feb 11;10:15. doi: 10.1186/1476-4598-10-15 (PMC3045364; doi:10.1186/1476-4598-10-15)
Supplement: Additional file 1 — Table S1. Assays on demand (Applied Biosystems) used for the human RT-PCR. Table S1 gives an overview about the Assays on demand used for the RT-PCR on the human samples. Table S2. Assays on demand (Applied Biosystems) used for the murine RT-PCR. Table S2 gives an overview about the Assays on demand used for the RT-PCR on the murine samples. Table S3. Primary antibodies used for immunhistochemical staining. Table S3 gives an overview about the Antibodys used for the immunohistochemistry on the human tissue samples. The Table includes information about the dilution, the Company and the catalog number of the antibody. Table S4. 173 probe sets significantly changed between controls and DCIS/IDC in WAP-TNP-8 mice. Table S4 shows all the genes found to be differentially expressed between control mice and DCIS/IDC in the WAP-TNP8 mice. [file 1476-4598-10-15-S1.DOC]

**Table S1**

| Gene | Assay-on-Demand ID |
| --- | --- |
| MUC1 | Hs00159357_m1 |
| SPP1 | Hs00959010_m1 |
| RRM2 | Hs01072069_g1 |
| FOXM1 | Hs00153543_m1 |
| EXO1 | Hs01116190_m1 |
| DEPDC1 | Hs00854841_g1 |
| NUSAP1 | Hs01006195_m1 |

**Table S2**

| Gene | Assay-on-Demand ID |
| --- | --- |
| Muc1 | Mm00449604_m1 |
| Spp1 | Mm01611440_mH |
| Rrm2 | Mm01287170_g1 |
| Foxm1 | Mm00514924_m1 |
| Exo1 | Mm00516302_m1 |
| Depdc1 | Mm00780966_s1 |
| Nusap1 | Mm00505602_m1 |

**Table S3**

| Antibody | Type | dilution | Secondary Antibody | Company | Catalog number |
| --- | --- | --- | --- | --- | --- |
| MUC1 | Polyclonal | 1:50 | Rabbit | US Biological | E3414-16A |
| SPP1 | Polyclonal | 1:100 | Goat | Imgenex | IMG-3855 |
| RRM2 | Monoclonal | 1:100 | Mouse | Abnova | H00006241-M01 |
| FOXM1 | Polyclonal | 1:100 | Goat | Imgenex | IMX -3091 |
| DEPDC1 | Monoclonal | 1:100 | Mouse | abcam | Ab57591 |
| NUSAP1 | Polyclonal | 1:100 | Mouse | Abnova | H00051203-B01 |

**Table S4**

| **Probe Set ID** | **Gene Symbol** | **Gene Title** |
| --- | --- | --- |
| 1415810_at | Uhrf1 | ubiquitin-like, containing PHD and RING finger domains, 1 |
| 1415811_at | Uhrf1 | ubiquitin-like, containing PHD and RING finger domains, 1 |
| 1415849_s_at | Stmn1 | stathmin 1 |
| 1415945_at | Mcm5 | minichromosome maintenance deficient 5, cell division cycle 46 (S. cerevisiae) |
| 1416076_at | Ccnb1 /// Ccnb1-rs1 /// EG434175 /// LOC667005 | cyclin B1, related sequence 1 /// cyclin B1 /// predicted gene, EG434175 /// similar to cyclin B1 |
| 1416120_at | Rrm2 | ribonucleotide reductase M2 |
| 1416251_at | Mcm6 | minichromosome maintenance deficient 6 (MIS5 homolog, S. pombe) (S. cerevisiae) |
| 1416299_at | Shcbp1 | Shc SH2-domain binding protein 1 |
| 1416309_at | Nusap1 | nucleolar and spindle associated protein 1 |
| 1416558_at | Melk | maternal embryonic leucine zipper kinase |
| 1416664_at | Cdc20 | cell division cycle 20 homolog (S. cerevisiae) |
| 1416802_a_at | Cdca5 | cell division cycle associated 5 |
| 1416961_at | Bub1b | budding uninhibited by benzimidazoles 1 homolog, beta (S. cerevisiae) |
| 1417019_a_at | Cdc6 | cell division cycle 6 homolog (S. cerevisiae) |
| 1417323_at | Psrc1 | proline/serine-rich coiled-coil 1 |
| 1417450_a_at | Tacc3 | transforming, acidic coiled-coil containing protein 3 |
| 1417457_at | Cks2 /// LOC100039474 /// LOC100044764 | CDC28 protein kinase regulatory subunit 2 /// similar to Cyclin-dependent kinases regulatory subunit 2 (CKS-2) |
| 1417458_s_at | Cks2 /// LOC100039474 /// LOC100044764 | CDC28 protein kinase regulatory subunit 2 /// similar to Cyclin-dependent kinases regulatory subunit 2 (CKS-2) |
| 1417541_at | Hells | helicase, lymphoid specific |
| 1417587_at | Timeless | timeless homolog (Drosophila) |
| 1417618_at | Itih2 | inter-alpha trypsin inhibitor, heavy chain 2 |
| 1417910_at | Ccna2 | cyclin A2 |
| 1417911_at | Ccna2 | cyclin A2 |
| 1418026_at | Exo1 | exonuclease 1 |
| 1418175_at | Vdr | vitamin D receptor |
| 1418264_at | Cenpk | centromere protein K |
| 1418281_at | Rad51 | RAD51 homolog (S. cerevisiae) |
| 1418856_a_at | Fanca | Fanconi anemia, complementation group A |
| 1418919_at | Sgol1 | shugoshin-like 1 (S. pombe) |
| 1419153_at | 2810417H13Rik | RIKEN cDNA 2810417H13 gene |
| 1419513_a_at | Ect2 | ect2 oncogene |
| 1419735_at | Csn3 | casein kappa |
| 1419943_s_at | Ccnb1 | cyclin B1 |
| 1420369_a_at | Csn2 | casein beta |
| 1420627_a_at | Csn1s1 | casein alpha s1 |
| 1420683_at | Bnipl | BCL2/adenovirus E1B 19kD interacting protein like |
| 1421001_a_at | Car6 | carbonic anhydrase 6 |
| 1421546_a_at | Racgap1 | Rac GTPase-activating protein 1 |
| 1422430_at | Fignl1 | fidgetin-like 1 |
| 1422462_at | Ube2t | ubiquitin-conjugating enzyme E2T (putative) |
| 1422513_at | Ccnf | cyclin F |
| 1422814_at | Aspm | asp (abnormal spindle)-like, microcephaly associated (Drosophila) |
| 1423123_at | Rad54l | RAD54 like (S. cerevisiae) |
| 1423124_x_at | Rad54l | RAD54 like (S. cerevisiae) |
| 1423174_a_at | Pard6b | par-6 (partitioning defective 6) homolog beta (C. elegans) |
| 1423463_a_at | D2Ertd750e | DNA segment, Chr 2, ERATO Doi 750, expressed |
| 1423774_a_at | Prc1 | protein regulator of cytokinesis 1 |
| 1423775_s_at | Prc1 | protein regulator of cytokinesis 1 |
| 1423847_at | Ncapd2 | non-SMC condensin I complex, subunit D2 |
| 1423877_at | Chaf1b | chromatin assembly factor 1, subunit B (p60) |
| 1423920_at | Ncaph | non-SMC condensin I complex, subunit H |
| 1424046_at | Bub1 | budding uninhibited by benzimidazoles 1 homolog (S. cerevisiae) |
| 1424105_a_at | Pttg1 | pituitary tumor-transforming 1 |
| 1424118_a_at | Spc25 | SPC25, NDC80 kinetochore complex component, homolog (S. cerevisiae) |
| 1424128_x_at | Aurkb | aurora kinase B |
| 1424143_a_at | Cdt1 | chromatin licensing and DNA replication factor 1 |
| 1424278_a_at | Birc5 | baculoviral IAP repeat-containing 5 |
| 1424292_at | Depdc1a | DEP domain containing 1a |
| 1424511_at | Aurka | aurora kinase A |
| 1424629_at | Brca1 | breast cancer 1 |
| 1424766_at | Ercc6l | excision repair cross-complementing rodent repair deficiency complementation group 6 - like |
| 1424971_at | Ccdc99 | coiled-coil domain containing 99 |
| 1425121_a_at | BC006965 | cDNA sequence BC006965 |
| 1425815_a_at | Hmmr | hyaluronan mediated motility receptor (RHAMM) |
| 1426817_at | Mki67 | antigen identified by monoclonal antibody Ki 67 |
| 1427161_at | Cenpf | centromere protein F |
| 1427275_at | Smc4 | structural maintenance of chromosomes 4 |
| 1427707_a_at | Stil | Scl/Tal1 interrupting locus |
| 1428104_at | Tpx2 | TPX2, microtubule-associated protein homolog (Xenopus laevis) |
| 1428485_at | Car12 | carbonic anyhydrase 12 |
| 1429171_a_at | Ncapg | on-SMC condensin I complex, subunit G |
| 1429172_a_at | Ncapg | on-SMC condensin I complex, subunit G |
| 1430811_a_at | Nuf2 | NUF2, NDC80 kinetochore complex component, homolog (S. cerevisiae) |
| 1433408_a_at | Mcm10 | minichromosome maintenance deficient 10 (S. cerevisiae) |
| 1433543_at | Anln | anillin, actin binding protein (scraps homolog, Drosophila) |
| 1433893_s_at | Spag5 | sperm associated antigen 5 |
| 1434280_at |  |  |
| 1434437_x_at | Rrm2 | ribonucleotide reductase M2 |
| 1434695_at | Dtl | denticleless homolog (Drosophila) |
| 1434748_at | Ckap2 | cytoskeleton associated protein 2 |
| 1434767_at | C79407 | expressed sequence C79407 |
| 1434850_at | Iqgap3 | IQ motif containing GTPase activating protein 3 |
| 1435306_a_at | Kif11 | kinesin family member 11 |
| 1435945_a_at | Kcnn4 | potassium intermediate/small conductance calcium-activated channel, subfamily N, member 4 |
| 1436170_a_at | Csn1s2a | casein alpha s2-like A |
| 1436186_at | E2f8 | E2F transcription factor 8 |
| 1436707_x_at | Ncaph | non-SMC condensin I complex, subunit H |
| 1437313_x_at | Hmgb2 | high mobility group box 2 |
| 1437611_x_at | Kif2c | kinesin family member 2C |
| 1437716_x_at | Kif22 | kinesin family member 22 |
| 1438852_x_at | Mcm6 | minichromosome maintenance deficient 6 (MIS5 homolog, S. pombe) (S. cerevisiae) |
| 1439040_at | Cenpe | centromere protein E |
| 1439377_x_at | Cdc20 | cell division cycle 20 homolog (S. cerevisiae) |
| 1448191_at | Plk1 | polo-like kinase 1 (Drosophila) |
| 1448205_at | Ccnb1 /// Ccnb1-rs1 | cyclin B1, related sequence 1 /// cyclin B1 |
| 1448226_at | Rrm2 | ribonucleotide reductase M2 |
| 1448314_at | Cdc2a | cell division cycle 2 homolog A (S. pombe) |
| 1448466_at | Cdca5 | cell division cycle associated 5 |
| 1448627_s_at | Pbk | PDZ binding kinase |
| 1448752_at | Car2 | carbonic anhydrase 2 |
| 1449104_at | Upk3a | uroplakin 3A |
| 1449171_at | Ttk | Ttk protein kinase |
| 1449199_at | Muc1 | mucin 1, transmembrane |
| 1449207_a_at | Kif20a | kinesin family member 20A |
| 1449254_at | Spp1 | secreted phosphoprotein 1 |
| 1449431_at | Trpc6 | transient receptor potential cation channel, subfamily C, member 6 |
| 1449699_s_at | C330027C09Rik | RIKEN cDNA C330027C09 gene |
| 1449708_s_at | Chek1 | checkpoint kinase 1 homolog (S. pombe) |
| 1449877_s_at | Kifc1 /// LOC100044746 | kinesin family member C1 /// similar to Kifc1 protein |
| 1450196_s_at | Gys1 | glycogen synthase 1, muscle |
| 1450496_a_at | 2810433K01Rik | RIKEN cDNA 2810433K01 gene |
| 1450677_at | Chek1 | checkpoint kinase 1 homolog (S. pombe) |
| 1450692_at | Kif4 | kinesin family member 4 |
| 1450862_at | Rad54l | RAD54 like (S. cerevisiae) |
| 1450886_at | Gsg2 | germ cell-specific gene 2 |
| 1450920_at | Ccnb2 | cyclin B2 |
| 1451128_s_at | Kif22 | kinesin family member 22 |
| 1451246_s_at | Aurkb | aurora kinase B |
| 1451358_a_at | Racgap1 | Rac GTPase-activating protein 1 |
| 1451407_at | Igsf5 /// Pcp4 | Purkinje cell protein 4 /// immunoglobulin superfamily, member 5 |
| 1451424_at | Gabrp | gamma-aminobutyric acid (GABA-A) receptor, pi |
| 1451483_s_at | 1700054N08Rik | RIKEN cDNA 1700054N08 gene |
| 1451851_a_at | Csn3 | casein kappa |
| 1452040_a_at | Cdca3 | cell division cycle associated 3 |
| 1452242_at | Cep55 | centrosomal protein 55 |
| 1452305_s_at | Cenpn | centromere protein N |
| 1452314_at | Kif11 | kinesin family member 11 |
| 1452458_s_at | Ppil5 | peptidylprolyl isomerase (cyclophilin) like 5 |
| 1452459_at | Aspm | asp (abnormal spindle)-like, microcephaly associated (Drosophila) |
| 1452534_a_at | Hmgb2 | high mobility group box 2 |
| 1452954_at | Ube2c | ubiquitin-conjugating enzyme E2C |
| 1453107_s_at | 4933413G19Rik /// Foxm1 /// Pebp1 | forkhead box M1 /// phosphatidylethanolamine binding protein 1 /// RIKEN cDNA 4933413G19 gene |
| 1454694_a_at | Top2a | topoisomerase (DNA) II alpha |
| 1455990_at | Kif23 | kinesin family member 23 |
| 1420081_s_at | D2Ertd750e | DNA segment, Chr 2, ERATO Doi 750, expressed |
| 1428304_at | Esco2 | establishment of cohesion 1 homolog 2 (S. cerevisiae) |
| 1428480_at | Cdca8 | cell division cycle associated 8 |
| 1428481_s_at | Cdca8 | cell division cycle associated 8 |
| 1428827_at | Whsc1 | Wolf-Hirschhorn syndrome candidate 1 (human) |
| 1429095_at | Cenpp | centromere protein P |
| 1429658_a_at | Smc2 | structural maintenance of chromosomes 2 |
| 1429660_s_at | Smc2 | structural maintenance of chromosomes 2 |
| 1431087_at | Spc24 | SPC24, NDC80 kinetochore complex component, homolog (S. cerevisiae) |
| 1434734_at | E130016E03Rik | RIKEN cDNA E130016E03 gene |
| 1434789_at | Depdc1b | DEP domain containing 1B |
| 1435005_at | Cenpe | centromere protein E |
| 1435575_at | Kntc1 | kinetochore associated 1 |
| 1435938_at | Ckap2l | cytoskeleton associated protein 2-like |
| 1436239_at | Slc5a5 | solute carrier family 5 (sodium iodide symporter), member 5 |
| 1436723_at | Cenpi | centromere protein I |
| 1436808_x_at | Mcm5 | minichromosome maintenance deficient 5, cell division cycle 46 (S. cerevisiae) |
| 1436847_s_at | Cdca8 | cell division cycle associated 8 |
| 1437019_at | 2200001I15Rik | RIKEN cDNA 2200001I15 gene |
| 1437370_at | Sgol2 | shugoshin-like 2 (S. pombe) |
| 1437580_s_at | Nek2 | NIMA (never in mitosis gene a)-related expressed kinase 2 |
| 1438434_at | Arhgap11a | Rho GTPase activating protein 11A |
| 1439520_at | Dtl | denticleless homolog (Drosophila) |
| 1439695_a_at | Kif20b | kinesin family member 20B |
| 1441757_at | 1190002F15Rik | RIKEN cDNA 1190002F15 gene |
| 1443694_at | Rgs20 | regulator of G-protein signaling 20 |
| 1443933_at | Tc2n | tandem C2 domains, nuclear |
| 1447363_s_at | Bub1b | budding uninhibited by benzimidazoles 1 homolog, beta (S. cerevisiae) |
| 1452806_at | 1500016O10Rik | RIKEN cDNA 1500016O10 gene |
| 1452912_at | Dscc1 | defective in sister chromatid cohesion 1 homolog (S. cerevisiae) |
| 1453067_at | Apitd1 | apoptosis-inducing, TAF9-like domain 1 |
| 1453226_at | 3000004C01Rik | RIKEN cDNA 3000004C01 gene |
| 1454744_at | F630043A04Rik | RIKEN cDNA F630043A04 gene |
| 1455818_at | 4930427A07Rik | RIKEN cDNA 4930427A07 gene |
| 1455983_at | Cdca2 | cell division cycle associated 2 |
| 1456077_x_at | Cdc25c | cell division cycle 25 homolog C (S. pombe) |
| 1456280_at | Clspn | claspin homolog (Xenopus laevis) |
| 1457026_at | Liph | lipase, member H |
| 1458374_at | C79407 | expressed sequence C79407 |
